# Is It Time to Use Probiotics to Prevent or Treat Obesity?

**DOI:** 10.3390/nu10111613

**Published:** 2018-11-01

**Authors:** Andrea Brusaferro, Rita Cozzali, Ciriana Orabona, Anna Biscarini, Edoardo Farinelli, Elena Cavalli, Ursula Grohmann, Nicola Principi, Susanna Esposito

**Affiliations:** 1 Pediatric Clinic, Department of Surgical and Biomedical Sciences, Università degli Studi di Perugia, 06132 Perugia, Italy; andreabrux@libero.it (A.B.); rita.cozzali@gmail.com (R.C.); annabiscarini@libero.it (A.B.); edoardo.farinelli@gmail.com (E.F.); elena-cavalli@libero.it (E.C.); Nicola.principi@unimi.it (N.P.); 2 Pharmacology Section, Department of Experimental Medicine, Università degli Studi di Perugia, 06132 Perugia, Italy; ciriana.orabona@unipg.it (C.O.); Ursula.grohmann@unipg.it (U.G.)

**Keywords:** body weight, dysbiosis, gut microbiota, obesity, probiotics

## Abstract

In recent years, attention has been given to the role potentially played by gut microbiota in the development of obesity. Several studies have shown that in individuals with obesity, the gut microbiota composition can be significantly different from that of lean individuals, that faecal bacteria can exert a fundamental role in modulating energy metabolism, and that modifications of gut microbiota composition can be associated with increases or reductions of body weight and body mass index. Based on this evidence, manipulation of the gut microbiota with probiotics has been considered a possible method to prevent and treat obesity. However, despite a great amount of data, the use of probiotics to prevent and treat obesity and related problems remains debated. Studies have found that the probiotic effect on body weight and metabolism is strain specific and that only some of the species included in the *Lactobacillus* and *Bifidobacterium* genera are effective, whereas the use of other strains can be deleterious. However, the dosage, duration of administration, and long-term effects of probiotics administration to prevent overweight and obesity are not known. Further studies are needed before probiotics can be rationally prescribed for the prevention or treatment of obesity. Control of the diet and environmental and life-style factors that favour obesity development remain the best solution to problems related to weight gain.

## 1. Introduction

Obesity is a major public health problem whose prevalence tends to continuously increase. The World Health Organization has estimated that in the last 40 years, the prevalence of obesity nearly tripled, and in 2016, over 650 million people around the world, including several million infants and children, were obese [1]. The clinical relevance of obesity is enormous. An increased body weight is associated with the development of several severe chronic conditions, such as cardiovascular diseases, diabetes mellitus, musculoskeletal disorders, and various cancers. Each year, 28 million individuals worldwide die from the consequences of being overweight or obese [2]. Moreover, obesity leads to a substantial medical, social, and economic burden [3].

An imbalance between energy intake and energy expenditure in genetically susceptible individuals is considered the most important cause of obesity development. Environmental and lifestyle factors, such as an increased intake of energy-dense food and a reduction of physical activity, are frequently identified as the most common conditions that favour energy imbalance [4]. However, in recent years, particularly after the availability of high-throughput sequencing technologies and their integration with advanced analytical methods that allow rapid and accurate microbial identification [4], attention has been given to the role potentially played by gut microbiota. The gut microbiota consists of trillions of microorganisms and thousands of bacterial species that have specific functions in the host’s nutrient metabolism, xenobiotic and drug metabolism, maintenance of structural integrity of the gut mucosal barrier, immunomodulation, and protection against pathogens (Table 1) [5].

Several studies have shown that dysbiosis (i.e., qualitative and quantitative modifications of the gut microbiota composition) can be associated with the development of both intestinal and extra-intestinal disorders. Dysbiosis has been described in irritable bowel syndrome [6], inflammatory bowel disease [7,8], colorectal cancer [9,10], allergic diseases [11], non-alcoholic steatohepatitis [12,13], arteriosclerotic diseases [14,15], several neurologic diseases [16], and metabolic syndromes [17,18], most notably diabetes and obesity [19,20]. Regarding overweight and obesity, several studies have shown that in obese individuals, the gut microbiota composition can be significantly different from that of lean individuals, that faecal bacteria can exert a fundamental role in modulating energy metabolism, and that modifications of gut microbiota composition can be associated with increases or reductions of body weight and body mass index (BMI) [19,20].

Based on this evidence, manipulation of the gut microbiota with probiotics has been considered a possible method to prevent and treat obesity. Several studies carried out with single probiotics or with a mixture of more than one of these potentially protective bacteria have tried to evaluate the role of probiotics in modulating body weight [21,22,23,24]. Despite a great amount of data, the use of probiotics to prevent and treat obesity and related problems remains debated. This narrative review will discuss the main results of the most important studies presently available related to this issue. 

## 2. Gut Microbiota Modification in Obesity

The potential association between gut microbiota and obesity was initially suggested by a series of studies carried out in experimental animals. It was shown that germ-free mice were significantly leaner than conventionally raised animals [25], the gut microbiota composition of genetically obese mice was significantly different from that of wild siblings fed with the same diet [26], and the transplantation of microbiota from obese and lean mice to germ-free animals led to a general increase of total body fat, with greater increases in subjects receiving faeces of obese individuals [19]. Moreover, studies that performed transplantation of gut microbiota from adult humans into germ-free mice clearly demonstrated that obesity-related microbiota phenotypes could be transferred, definitively confirming that gut microbiota play a crucial role in conditioning body weight and fat deposition [27,28].

When attempts to detail the differences in gut microbiota composition between obese and lean individuals were made, several studies reported that, in comparison to lean subjects, individuals with obesity were characterized by a different prevalence of the two bacteria divisions, *Firmicutes* and *Bacteroidetes*, which are the most common bacteria types in the gut microbiota [6,19]. Moreover, people with obesity frequently had a lower bacterial diversity. However, the data collected from animals were not always comparable to those obtained from humans. In overweight and obese animals, Bacteroidetes were systematically reduced and Firmicutes were frequently increased, leading to a higher Firmicutes to Bacteroides (F/B) ratio [19]. Moreover, it was found that similar modifications could be obtained by gut microbiota manipulation through the diet or the administration of antibiotics [19,29].

Based on these studies, it could have been concluded that the F/B ratio could be considered a marker of obesity and that attempts to bring this ratio back to normal could allow prevention and treatment of obesity. However, in humans, not all of the studies reported similar results. Although in most cases it was shown that the reduction in the abundance of *Bacteroidetes* and a lower diversity of gut flora were associated with obesity, some studies could not demonstrate this association, or the opposite trend was found [30,31,32,33,34,35,36]. Moreover, two recent meta-analyses that were specifically planned to evaluate the hypothesis that variations in the gut microbiota could explain or be used to predict obesity status in humans did not find a clear trend between the F/B ratio and obesity. Walters et al. [37] concluded that although in all studies except one [38] a trend showing an increase in the F/B ratio in obese compared to lean subjects was shown, no significant differences overall between obese and lean categories were found. Moreover, no consistent alpha diversity trend in gut microbiota composition according to BMI was demonstrated. Sze and Schloss, by using 10 independent studies, reported that although an association between gut microbiota composition and obesity could be demonstrated, it was smaller than can be detected by most microbiome studies [39]. Furthermore, it was calculated that in a single subject, the accuracy of the microbiota composition for the prediction of obesity status was between 33.0% and 64.8%. Taken together, these findings indicate that the increase in the F/B ratio is too rough of an indicator of microbial variations to be relied on for the prediction of being overweight or obese. Additionally, it seems clear that only studies considering more in-depth microbial evaluations might indicate which bacterial genera and/or species are strictly related to weight modulation and obesity development. However, the presently available data in this regard are limited, and firm conclusions cannot yet be drawn.

Evidence of an association between the prevalence of a given bacterial genus or species and obesity or lean status does not explain whether the microorganism(s) is(are) truly the cause of obesity or normal weight or, in contrast, whether the emergence or disappearance of obesity is simply the consequence of external factors such as diet. However, the genera *Bifidobacterium*, *Oscillospira*, *Erwinia, Succinivibrio*, and *Alistipes* were considered protective because they are usually found to be more abundant in subjects with normal weight than in obese individuals [40,41]. On the other hand, *Enterobacter* [42] and *Bacteroides* [34] were prominent in obese individuals and are considered to be microbial factors that favour obesity development. However, for some bacterial genera, conflicting results were reported. For example, this is the case for the genus *Lactobacillus*, whose concentrations were found to progressively decline in adolescents following a weight-loss programme, suggesting a protective effect [43], but were higher in overweight and obese children than in healthy controls, suggesting a contribution to obesity [44]. Regarding bacterial species, a protective effect was ascribed to *Bacteroides faecichinchillae*, *Bacteroides thetaiotaomicron*, *Blautia wexlerae*, *Clostridium bolteae*, *Flavonifractor plautii* and *Akkermansia muciniphila* [45,46]. In contrast, *Blautia hydrogenotorophica*, *Coprococcus catus*, *Eubacterium ventriosum*, *Ruminococcus bromii*, and *Ruminococcus obeum* have been associated with the development of obesity [45]. Data regarding some species contrast with those regarding the whole genus (e.g., *Bacteroides*), clearly confirming how difficult it can be to identify the bacteria that are truly associated with obesity if the analysis of the gut microbiota is not extremely detailed. 

## 3. Regulation of Body Weight by Gut Microbiota

Knowledge of how the gut microbiota can modulate body weight and energy metabolism is essential to understand which intestinal bacteria have a major role in this regard. Three primary mechanisms—short-chain fatty acid (SCFA) production, regulation of bile acid metabolism, and induction/protection from metabolic endotoxaemia—play a major role.

### 3.1. Short-Chain Fatty Acid Production

It is known that gut microbiota ferment non-digestible polysaccharides, thereby producing a large amount of SCFAs, mainly acetate, propionate, and butyrate [47]. SCFAs represent an important energy source—constituting nearly 10% of the daily energy supply in omnivores [48]—and can be found in greater concentrations in faecal samples of obese adults and children compared to normal weight controls [35]. SCFAs interact with the G-protein-coupled receptors Gpr41 and Gpr43, which are expressed on gut epithelial cells [49], inducing the production of peptide YY (PYY) and glucagon-like peptide one (GLP-1). These two gut hormones reduce gut motility [50], promote satiety, and suppress energy intake [51]. Finally, SCFA-Gpr41 and -Gpr43 interactions stimulate leptin production [52] and profoundly affect inflammatory responses [53] that are responsible for the development of obesity-related metabolic disturbances such as insulin resistance, lipogenesis, and increased triglyceride stores [54].

However, not all of the gut microbiota components have the same ability to ferment non-digestible polysaccharides. Moreover, the qualitative and quantitative production of SCFAs can significantly vary from agent to agent and differ according to the type of substrate. Finally, different SCFAs can have different metabolic properties. Whereas acetate production is common to several bacterial groups, propionate and butyrate production seem to be more highly conserved and substrate specific. Propionate is dominated by relatively few bacterial genera, among which *Akkermansia municiphila* is the most important [55]. Fermentation of fucose and rhamnose leads to propionate, whereas that of resistant starch, mainly due to *Ruminococcus bromii,* produces butyrate [55]. Butyrate is also produced by *Faecalibacterium prausnitzii*, *Eubacterium rectale*, *Eubacterium hallii* and *Ruminococcus bromii* [55].

Regarding functions of the various SCFAs, they are not completely defined, and in some cases conflicting data are reported. For years it was thought that increased acetate production was a driver of metabolic syndrome [56]. On the contrary, it has recently been shown that rather than having detrimental effects, the availability of a large amount of acetate can be beneficial, as it is associated with the reduction of appetite, a marked reduction of lipid accumulation in adipose tissue, protection against the accumulation of fat in the liver, and improves glucose tolerance [57,58].

Propionate is associated with significant systemic metabolic effects, as it is promptly absorbed and can be found in high concentrations in the circulation. It has been shown that propionate increases the levels of PYY, GLP-1, and leptin, decreases serum cholesterol levels and liver lipogenesis, and induces satiety, thereby strongly contributing to weight control. Propionate also appears able to exert a significant protective effect in the gut, reducing the risk of cancer development [59]. 

Butyrate, which is mainly used by colonocytes as an energy source and is poorly detected in the circulation [60], exerts strong anti-infective and anti-inflammatory properties at gut level [61]. Moreover, it appears able to prevent increases in body weight without altering food intake or energy expenditure, improves insulin sensitivity, as measured by glucose and insulin tolerance tests, and decreases the respiratory exchange ratio [62].

### 3.2. Regulation of Bile Acid Metabolism

Gut microbiota deconjugate bile acids through the action of microbial enzymes. Bacterial bile salt hydrolases (BSH) are produced by various microbial species, including those in the *Lactobacillus, Bifidobacterium, Enterococcus*, and *Clostridium* genera [63]. The activity of microbial BSH can, however, vary from species to species. Deconjugation may be protective for some bacteria, including *Lactobacillus* and *Bifidobacterium* genera, as free bile acids disrupt their membrane integrity [64]. However, the resistance of various bacterial species can be significantly different. Deconjugated bile acids are poorly absorbed and are mainly excreted with the faeces. This can reduce serum cholesterol levels because de novo bile acid synthesis from cholesterol is stimulated, as evidenced by animal studies [65]. 

Moreover, increased or reduced BSH activity might lead to similar variations of body weight through nuclear farnesoid X receptor (FXR)- and G-protein-coupled bile acid receptor 1 (TGR5)-mediated mechanisms. FXR regulates the synthesis, transport, and enterohepatic circulation of bile acids by modulating the expression of related genes in the liver and small intestine. The gut microbiota influence on FXR activity appears to be critical for body weight increase and glucose and lipid homeostasis [66]. FXR-deficient animals are protected from obesity, and pharmacologic inhibition of FXR activity is considered a potential method to reduce obesity and treat related metabolic disturbances [63]. Similar to FXR, TGR5 is a metabolic regulator involved in energy homeostasis, bile acid homeostasis, and glucose metabolism [67]. In obese mice, it has been shown that TGR5 signalling induces intestinal glucagon-like peptide-1 (GLP-1) release, leading to improved liver and pancreatic function and enhanced glucose tolerance [68]. Among gut beneficial bacteria, a relevant role in regulating bile acid homeostasis and functions seems to be exerted by *Akkermansia muciniphila* [69]. 

### 3.3. Induction of Metabolic Endotoxaemia

Increased plasma lipopolysaccharide (LPS) concentrations are associated with metabolic derangements, including metabolic endotoxaemia. LPS is the major glycolipid component of the outer membrane of Gram-negative bacteria, which constitute approximately 70% of the gut microbiota [70]. When Gram-negative bacteria increase, accompanied by a reduction of *Lactobacillus* spp., *Bifidobacterium* spp., and *Bacteroides-Prevotella* spp., as occur after a high-fat diet, gut permeability increases. LPS derived from bacterial lysis is significantly absorbed, and various pro-inflammatory pathways and increased oxidative stress is activated [71]. 

Malfunctioning of intestinal mucosa is mediated by the linkage of LPS to toll like receptor (TLR)-4 and the subsequent activation of the nuclear factor kappa-light-chain-enhancer of activated B cells (NFκB). NFkB is a protein complex that is contained in almost all cell types and controls DNA transcription, cytokine production and cell survival [72]. Its activation leads to chronic intestinal inflammation and metabolic endotoxaemia with the emergence of glucose intolerance, hepatic insulin resistance, and fat accumulation. The strict relationship between high-fat diet-mediated changes in the gut microbiota and metabolic endotoxaemia is confirmed by the evidence that the transplantation of an endotoxin-producer bacterium, *Enterobacter cloacae* B29, from obese individuals to germ-free mice on a high-fat diet was associated with the development of obesity and insulin resistance. In contrast, animals on a normal chow diet did not show any metabolic disturbance [42]. 

## 4. Use of Probiotics to Prevent or Treat Overweight and Obesity

### 4.1. Studies in Experimental Animals

Most of the studies carried in experimental animals have clearly demonstrated that administration of probiotics can be effective in the prevention and treatment of obesity. Moreover, it is shown that the benefits for body weight are frequently associated with favourable metabolic effects. However, not all probiotics have the same activity; it was found that the impacts on body weight, fat mass, glucose metabolism, inflammatory markers, plasma and hepatic lipids, or plasma cholesterol levels are strictly species and strain specific [73,74], and in some cases, they can induce a paradoxical effect favouring weight gain [75]. 

*Lactobacillus* and *Bifidobacterium* species are the most studied probiotics. *Lactobacillus curvatus* HY7601 and *Lactobacillus plantarum* KY1032 administered alone or in combination for 9 weeks to mice were found to limit fat accumulation in adipose tissue and liver. In addition, the treated animals showed a marked reduction of cholesterol in the plasma and liver. Interestingly, the combination was found to be more effective in inhibiting the gene expression of various enzymes related to fatty acid synthesis and oxidation in the liver compared to the single strains [76]. When the same bacterial combination or placebo was given for 10 weeks to obese mice maintained on a high-fat diet, the animals receiving probiotics had an increase in weight that was 38% lower than those given placebo. Compared to controls, the treated animals had a significant reduction of plasma cholesterol (17%, *p* < 0.05), plasma leptin (49%, *p* = 0.048) and insulin (67%, *p* = 0.025) [77]. Finally, a significant modification in the gut microbiota composition occurred. *Lactobacillus* and *Bifidobacterium* genera increased and *Clostridiaceae*, *Akkermansia*, and *Escherichia coli* decreased [77]. 

Similar results were obtained when *Lactobacillus paracasei* CNCM I-4270 and *Lactobacillus rhamnosus* CNCM I-3690 were used [78]. Although with slight differences, both probiotics significantly attenuated high-fat diet-induced weight gain, improved glucose–insulin homeostasis, and reduced hepatic steatosis. They also reduced the infiltration of pro-inflammatory (CD11c+, MMP-12+) macrophages into adipose tissue. As macrophage infiltration is an underlying cause of chronic adipose inflammation, insulin resistance and other obesity complications [17], this finding is consistent with the improvement of glucose homeostasis and the reduction of fatty liver disease seen in probiotic-treated animals. Finally, both probiotics induced significant modifications of the gut microbiota composition, particularly an increase in *Barnesiella* with a relevant amount of faecal acetate. 

The modulation of body weight and a positive impact on energy metabolism were also reported for other *Lactobacillus* species. The administration of *Lactobacillus rhamnosus* GG for 13 weeks to mice fed with a high-fat diet resulted in a reduced liver, mesenteric, and subcutaneous adipose tissue weight compared to non-treated controls [79]. Similarly, the serum levels of triglycerides and cholesterol were also significantly reduced in treated mice. It was shown that the reduction of fat accumulation was mediated by the downregulated expression of lipogenic and pro-inflammatory genes in the liver. Similarly, the reduction in cholesterol was associated with lower and higher expression levels of genes involved in cholesterol synthesis and cholesterol efflux, respectively. 

The administration of skimmed milk fermented by *Lactobacillus gasseri* SBT2055 to lean rats for 4 weeks resulted in lowering the mesenteric adipose tissue weight (23%; *p* < 0.05), adipocyte size (28%; *p* < 0.001), and serum leptin concentration (36%; *p* < 0.05) compared to controls. Diabetic rats fed with yoghurt supplemented with *Lactobacillus acidophilus* and *Lactobacillus casei* had a marked reduction of hyperglycaemia and hyperinsulinaemia [80,81]. Positive results were also obtained with several *Bifidobacterium* strains [73,82,83]. However, it was shown that the impact of the different *Bifidobacterium* species on the gut microbiota, weight changes, and metabolic markers can vary from species to species. Yin et al. compared the effects of four *Bifidobacterium* strains (*Bifidobacterium* L66-5, L75-4, M13-4 and FS31-12) in an obese murine model induced by a high-fat diet [75]. These authors found that whereas *Bifidobacterium* L75-4 and FS31-12 had no effect on weight gain, lipid metabolism, or glucose metabolism, *Bifidobacterium* M13-4 improved body weight gain (264.27 ± 26.91 vs. 212.55 ± 18.54, *p* = 0.001), while *B*. L66-5 induced a decrease in BW (188.47 ± 11.96 vs. 212.55 ± 18.54, *p* = 0.043). Surprisingly, all four strains reduced serum and liver triglycerides and significantly alleviated lipid deposition in the liver. The effects of *Bifidobacterium* species can be different from those of *Lactobacillus* species. In the study by Wang et al., in which *Bifidobacterium animalis* subsp. *lactis* I-2494 was compared with *Lactobacillus paracasei* CNCM I-4270 and *Lactobacillus rhamnosus* CNCM I-3690, it was shown that all three probiotic strains had a positive effect, but this was associated with a distinct modification of gut microbiota and with a strain-specific reduction of obesity complications [78]. Contrary to *Lactobacillus* strains, which did not have any effect in this regard, *Bifidobacterium* strain administration was associated not only with an increased concentration of this probiotic in faeces but also with a significant reduction of adipose and hepatic tumor necrosis factor (TNF)-a gene expression and with a lower circulating LPS load. 

Other probiotics with a demonstrated anti-obesity effect are *Bacteroides uniformis* CECT 7771 [84], *Pediococcus pentosaceus* LP28 [85], and *Saccharomyces boulardii* [86]. However, the most attractive probiotic is *Akkermansia muciniphila*. This is a mucin-degrading agent that resides in the mucus layer and is found in lower concentrations in obese individuals [87]. The administration of *Akkermansia muciniphila* for 4 weeks to obese and diabetic mice, without changes in food intake, resulted in a relevant reduction of body weight and diabetes markers [88]. Moreover, it was shown that there was a strict correlation between gut concentrations of this bacterium and gut inflammation and permeability [89]. An abundance of *Akkermansia muciniphila* was associated with decreased metabolic endotoxaemia and adipose tissue inflammation due to the restoration of the original mucus layer and specific antimicrobial peptides. Finally, this bacterium was demonstrated to be able to modulate the cannabinoid system and to increase the production of propionate [90]. However, all the conclusions drawn from these studies must be evaluated with caution because of some concerns on good laboratory practices. Methods used to evaluate probiotic impact on animal body weight significantly varied form study to study. Animals of different age fed with different diets were frequently used. Moreover, dose of probiotics and duration of administration were not uniform. All these factors could explain the contradictory literature. 

### 4.2. Studies in Humans

Based on studies carried out in experimental animals, most of the preparations used in clinical trials that have planned to evaluate the impact of probiotics on weight changes contained strains of the genera *Lactobacillus* and *Bifidobacterium*. A great number of studies are available [77,91,92,93,94,95,96,97,98,99,100] and in most of them, positive results are reported in both adults and children. For example, Kadooka et al. conducted a multicentre, double-blind, randomized, placebo-controlled trial in adults with a higher BMI and abdominal visceral fat area, and they administered fermented milk containing *Lactobacillus gasseri* LG2055 or only fermented milk for 12 weeks [91]. In the subjects receiving the probiotic, there was a 4.6% and a 3.3% decrease in the abdominal visceral and subcutaneous fat areas, respectively. Body weight was reduced by 1.4% and BMI by 1.5%. By contrast, none of these parameters changed in the controls. The administration of *Lactobacillus rhamnosus* GG to pregnant women during four weeks of pregnancy and to the child during the first 6 months of life was associated with a reduced weight gain in later life, at least until the end of the fourth year [100].

However, the majority of the studies were of poor quality, from which firm conclusions cannot be drawn. Moreover, in the studies with a limited risk of bias, the sample size was generally small, but significant differences in the type and dosage of the prescribed probiotic, treatment duration, and feeding type can be demonstrated. The natural modification of the gut microbiota composition during the first periods of life and the role of external factors such as diet and antibiotic consumption in inducing dysbiosis are poorly or not at all considered. Additionally, the results are frequently conflicting. All these factors explain why pooling data to perform meta-analyses is difficult, and conclusions of the different meta-analyses significantly vary.

Park and Bae carried out a meta-analysis of the studies regarding the use of probiotics for weight loss that were published up until December 28, 2014, excluding those enrolling pregnant women and infants [101]. Initially, 368 articles were selected. However, only 9 were randomized controlled trials (RCTs), and only 4 could be included in the meta-analysis, as only these studies provided means and SDs for body weight. A total of approximately 100 subjects were treated with probiotics and 100 received placebo. Changes in body weight, BMI, and, when possible, visceral fat mass were studied. No significant difference between groups was observed. The difference in mean visceral fat mass was also quite similar. The authors concluded that probiotics were ineffective in controlling weight changes. Similar conclusions were drawn by Borgeraas et al. in a similar meta-analysis [102]. On the other hand, substantially different results were reported by a more recent meta-analysis in which studies published up until August 2017 regarding the treatment of overweight and obese adults were considered [103]. Among a total of 8009 identified studies, 21 randomized controlled trials were analysed. It was shown that probiotic use was associated with a significant reduction in all the studied parameters (i.e., body weight and fat mass). However, data regarding the impact of the probiotic dosage were conflicting. Low doses were associated with a lower BMI reduction but with a greater fat mass decrease. Finally, a longer duration of probiotic use, even with low-dose administration, led to a significant reduction of both weight and BMI. The study’s conclusions were that dietary agents for the modulation of the gut microbiome are essential tools for obesity treatment.

Still, different conclusions were drawn by the authors of another meta-analysis in which RCTs and crossover RCTs were considered and results were stratified by age [104]. Studies regarding pregnant women, preterm babies, neonates, and subjects with gastrointestinal problems that might mask the effects of microbiota modulation were excluded. Starting from more than 1000 articles, 35 studies (14 in adults, 7 in children and 14 in infants) were considered to have a relatively low risk of bias and were analysed.

In adults, the use of various *Lactobacillus* strains (2.7 × 10^10^ cfu/day of probiotic administration for 2–3 months) was associated with significant weight loss. The magnitude of weight reduction varied from study to study, but these variations were not considered to be dependent on the type of probiotic used, intervention duration or characteristics of the baseline population. In children, the meta-analysis demonstrated a significant increase in the weight of subjects receiving probiotics (mainly *Lactobacillus* species) compared to the controls. Similar results were found in infants receiving a probiotic-enriched formula from the age of 3 weeks to 10 months. In this case, an increase in weight with borderline significance was shown. Further data showing a potential positive effect on weight gain from probiotic use have been recently reported. Jones et al. conducted a double-blind, randomized, placebo-controlled trial in 19 obese adolescents, administering three packets per day of a mixture of *Lactobacillus* species (*Lactobacillus. acidophilus* BA05, *Lactobacillus plantarum* BP06, *Lactobacillus paracasei* BP07, *Lactobacillus delbrueckii* subsp. *bulgaricus* BD08), *Bifidobacterium* species (*Bifidobacterium breve* BB02, *Bifidobacterium longum* BL03, *Bifidobacterium infantis* BI04) and *Streptococcus thermophilus* BT01 for 16 weeks [105]. Compared to placebo, the adolescents who had received the probiotics had significantly increased adiposity and trunk, with no significant effects on gut microbiota, gut appetite-regulating hormones, liver fat and fibrosis, or dietary intake [19]. 

## 5. Conclusions

The association of dysbiosis with obesity and related metabolic problems has been shown in both animals and humans. However, which components of the gut microbiota are the cause of weight gain and abnormal glucose and fat metabolism and which are protective against obesity and metabolic derangement is currently imprecisely defined. Some information can be derived from the analysis of the composition of the gut microbiota in obese individuals and from studies that have evaluated the impact of the various intestinal bacteria on fat and glucose metabolism. Strains included within the *Lactobacillus* and *Bifidobacterium* genera and *Akkermansia muciniphila* are those for which the most attractive results have been obtained. However, several studies have found that the probiotic effect on body weight and metabolism is strain specific and that only some of the species included in the *Lactobacillus* and *Bifidobacterium* genera are effective, whereas the use of other strains can be deleterious. However, the identification of strains that are potentially associated with a beneficial effect is not enough to suggest their systematic use in the treatment of obesity and related metabolic disturbances. The dosage, duration of administration and long-term effects of the administration of the different strains are not known. Further studies are needed before probiotics can be rationally prescribed for the prevention or treatment of obesity. Control of the diet and environmental and life-style factors that favour obesity development remain the best solution to problems related to weight gain.

## Figures and Tables

**Table 1:** Microbiota functions.

Function	Brief Explanation
Metabolite production	The fermentation of complex carbohydrates results in the production of short-chain fatty acids (SCFAs), which are involved in many cellular processes and metabolic pathways, in the enhancement of the gut barrier function and in the regulation of immune system and inflammatory responses.
Vitamin production	Microbiota synthesize essential vitamins that humans cannot produce (e.g., vitamin B12, vitamin K); a dysregulation results in metabolic pathologies such as obesity and type 2 diabetes mellitus.
Influence on epithelial homeostasis	Microbiota promote epithelial integrity by influencing the turnover of epithelial cells and modulating mucus properties.
Development of the immune system	Both intestinal mucosal defenses and the systemic immune system are modulated by microbiota, resulting in a greater protection against infections and against inflammatory diseases.
Influence on pathogen colonization	Microbiota compete with pathogens for attachment sites and nutrients, and they produce antimicrobial substances.
